# Antidiabetic and Renoprotective Effects of *Cladophora glomerata* Kützing Extract in Experimental Type 2 Diabetic Rats: A Potential Nutraceutical Product for Diabetic Nephropathy

**DOI:** 10.1155/2015/320167

**Published:** 2015-03-26

**Authors:** Chutima Srimaroeng, Atcharaporn Ontawong, Naruwan Saowakon, Pornpun Vivithanaporn, Anchalee Pongchaidecha, Doungporn Amornlerdpison, Sunhapas Soodvilai, Varanuj Chatsudthipong

**Affiliations:** ^1^Department of Physiology, Faculty of Medicine, Chiang Mai University, Chiang Mai 50200, Thailand; ^2^Division of Physiology, School of Medical Sciences, University of Phayao, Phayao 56000, Thailand; ^3^School of Anatomy, Institute of Science, Suranaree University of Technology, Nakhon Ratchasima 30000, Thailand; ^4^Department of Pharmacology, Faculty of Science, Mahidol University, Bangkok 10400, Thailand; ^5^Faculty of Fisheries Technology and Aquatic Resources, Maejo University, Chiang Mai 50290, Thailand; ^6^Department of Physiology, Faculty of Science, Mahidol University, Bangkok 10400, Thailand

## Abstract

*Cladophora glomerata* extract (CGE) has been shown to exhibit antigastric ulcer, anti-inflammatory, analgesic, hypotensive, and antioxidant activities. The present study investigated antidiabetic and renoprotective effects of CGE in type 2 diabetes mellitus (T2DM) rats. The rats were induced by high-fat diet and streptozotocin and supplemented daily with 1 g/kg BW of CGE for 12 weeks. The renal transport function was assessed by the uptake of *para*-aminohippurate mediated organic anion transporters 1 (Oat1) and 3 (Oat3), using renal cortical slices. These two transporters were known to be upregulated by insulin and PKC*ζ* while they were downregulated by PKC*α* activation. Compared to T2DM, CGE supplemented rats had significantly improved hyperglycaemia, hypertriglyceridemia, insulin resistance, and renal morphology. The baseline uptake of *para*-aminohippurate was not different among experimental groups and was correlated with Oat1 and 3 mRNA expressions. Nevertheless, while insulin-stimulated Oat1 and 3 functions in renal slices were blunted in T2DM rats, they were improved by CGE supplementation. The mechanism of CGE-restored insulin-stimulated Oat1 and 3 functions was clearly shown to be associated with upregulated PKC*ζ* and downregulated PKC*α* expressions and activations. These findings indicate that CGE has antidiabetic effect and suggest it may prevent diabetic nephropathy through PKCs in a T2DM rat model.

## 1. Introduction

*Cladophora glomerata* (CG) Kützing has been naturally grown in the north of Thailand. It is commonly known in Thai as “Kai.” Local people have traditionally used this alga as an ingredient in several northern Thai dishes and believed that it could serve as a medicinal plant for several diseases [1]. Previous* in vivo* studies have suggested that CG extract (CGE) exhibited antigastric ulcer, anti-inflammatory, analgesic, hypotensive, and antioxidant activities* in vitro *and* in vivo* [1]. Despite the wide range of supposed remedies, empirical evidence of an efficacious effect of CGE on a particular disease has not been reported.

Type 2 diabetes mellitus (T2DM) is a metabolic disorder that can be characterized by hyperglycaemia, insulin resistance, and/or relative insulin deficiency [2]. Prolonged hyperglycaemia can induce mitochondrial dysfunction and results in an enhanced reactive oxygen species (ROS) or oxidative stress production that could develop diabetic complications such as retinopathy, peripheral neuropathy, and nephropathy. Oxidative stress induced diabetic complications could also similarly be exacerbated by elevated free fatty acids that increased mitochondrial uncoupling and *β*-oxidation, as well as depleted endogenous antioxidants by reducing intracellular glutathione [3, 4]. This adverse oxidative stress was implicated to be a potential factor for vascular and renal tubular damage in diabetic nephropathy by increasing the productions of sorbitol, prostanoids, transforming growth factor-*β* (TGF-*β*), vascular endothelial growth factor (VEGF), and advanced glycation end-product (AGE) and also by activating common stress-signaling mediators, nuclear factor-*κ*B (NF*κ*B), and protein kinase C (PKC) [4–6]. Moreover, the molecular mechanism underlying the structural pathogenesis of diabetic nephropathy has also been proposed to be involved with several cellular events. For instance, hyperglycaemia induced the productions of AGE, PKC, and NF*κ*B and then stimulated the activities of TGF-*β* in both renal tubular and mesangial cells as well as VEGF in podocytes, followed by the various extracellular matrix (ECM) syntheses including collagen types I and IV and fibronectin [5, 6]. These consequences could then trigger the phenotypic transitions of mesangial and tubular epithelial cells to glomerular and tubulointerstitial fibrosis, respectively, where these have been observed in diabetic nephropathy [7].

The kidney plays a crucial role in the secretion of endogenous and exogenous compounds and also their metabolites by various membrane transporters. Thus, the changes in expressions and/or functions of renal transporters could also affect their substrate concentrations and pharmacokinetics [8, 9]. At present, the renal membrane transporters have been cloned and identified [8, 9], and, among these, organic anion transporters 1 (Oat1: SLC22A6) and 3 (Oat3: SLC22A8) are the highest expression [10] on the basolateral membrane and play the primary role in organic anion clearance from blood circulation to renal tubular epithelial cells, resulting in excretion of organic anions into tubular lumen. Both Oat1 and 3 recognize a broad spectrum of substrates and mediate with high-affinity transport of several organic anion compounds, including endogenous prostaglandin E2 and F2*α*, antiviral adefovir, nonsteroidal anti-inflammatory drugs, ochratoxin A, indoxyl sulfate, and* para*-aminohippurate (PAH), which is known to be a typical organic anion substrate for Oat1 and 3 [8, 11]. Recent studies have demonstrated that renal transporter expressions and/or functions could be influenced under certain pathological status, leading to accumulation of these therapeutic drugs or xenobiotic substrates into proximal tubular cells and obliteration of renal secretory function. For examples, the tubular cationic clearance was significantly reduced in streptozotocin- (STZ-) induced diabetic rats, suggesting that a decrease of tubular cationic clearance was due to downregulation of basolateral organic cation transporters 1 (Oct1), 2 (Oct2), and/or 3 (Oct3) expressions [12]. A decreased PAH transport mediated by Oat1 and 3 transporters was also shown in type 1 diabetes mellitus (T1DM) rats and mice, and insulin treatment was able to restore their functions [13, 14]. Moreover, progressive chronic renal failure (CRF) mimicked by 5/6 nephrectomy has been extensively reviewed to associate with a decrease of PAH clearance and/or Oat1, 2, and 3 expressions in rats [15]. More recently, kidney biopsy specimens from patients proved diabetic nephropathy have revealed a marked reduction of Oat1 and 3 mRNA expressions in parallel with a significant decrease of urinary organic anion metabolite, a homovanillic acid. This indicated that renal Oat1 and 3 in proximal tubules were influenced by certain pathological status and strongly correlated with the progression of diabetic kidney disease [16].

Several studies have also demonstrated that specific protein kinases could differentially modulate Oat1 and 3 transports of organic anions. The activated PKC*α* by angiotensin II downregulated Oat3 function, and GÖ6976, a specific PKC*α* inhibitor, reversed this inhibitory effect [17]. On the other hand, either insulin or EGF was able to increase rOat1 and 3 transports of organic anions through PKC*ζ* activation, and a specific PKC*ζ* inhibitor, namely, PKC*ζ* pseudosubstrate (PKC*ζ*-PS), could blunt PKC*ζ* activation and thereby reduce the effect of insulin or EGF on stimulation of rOat1 and 3 functions [18]. More recently, we have also shown that polyphenol-rich* Spirogyra neglecta* alga extract was able to improve renal oxidative stress and maintain renal organic anion transport function mediated by Oat3 in T2DM rats [19]. Therefore, improved Oat1 and/or 3 functions could delay or prevent the progression of renal diseases. Thus, this study aimed to investigate whether the CGE could improve T2DM condition and restore renal organic anion transport function, which may delay or prevent the progression of diabetic nephropathy. The chemical constituents of CGE and the mechanisms by which CGE restored renal organic anion transport function in T2DM rat model were also identified. These findings could prove insightful for developing CGE into potential nutraceutical or pharmaceutical products for diabetes.

## 2. Materials and Methods

### 2.1. Chemicals

Polyclonal rabbit anti-PKC*α* and phosphorylated PKC*α* (p-PKC*α*) were purchased from Santa Cruz (CA, USA) while monoclonal mouse anti-lamin B1 and polyclonal rabbit anti-phosphorylated PKC*ζ* were purchased from Cell Signaling (Danvers, MA, USA). Monoclonal mouse anti-*β* actin was obtained from Abcam (Cambridge, MA, USA) and monoclonal mouse anti-Na^+^-K^+^-ATPase was obtained from Novus Biological (Littleton, CO, USA). Polyclonal rabbit anti-PKC*ζ* was obtained from Zymed (Invitrogen, Carlsbad, CA, USA). Tritiated* para*-aminohippurate ([^3^H]-PAH; specific activity (SA) 1 Ci/mmol) was obtained from PerkinElmer Life Sciences (Boston, MA, USA). Streptozotocin (STZ) and CelLytic MT mammalian tissue lysis/extraction reagent were purchased from Sigma Aldrich (St. Louis, MO, USA). Metformin was obtained from Merck (Darmstadt, Germany). Insulin was obtained from Biocon (Bangkok, Thailand), and protease inhibitor was purchased from Roche Diagnostics (Indianapolis, IN, USA). Phorbol 12-myristate 13-acetate (PMA), GÖ6976, and PKC*ζ* pseudosubstrate (PKC*ζ*-PS) were purchased from Calbiochem (EMD Millipore, MA, USA). All other chemicals with high purity were obtained from commercial sources.

### 2.2. *Cladophora glomerata* Extract Preparation, Purification, and Qualification

The identification of species of* Cladophora glomerata *extract (CGE) was confirmed and kindly provided by Dr. Yuwadee Peerapornpisal, Faculty of Science, Chiang Mai University. A voucher specimen (number AARL G048) has been deposited at the herbarium of the Applied Algal Research Laboratory, Department of Biology, Faculty of Science, Chiang Mai University, Chiang Mai, Thailand. Dried CGE was weighed and blended thoroughly followed by boiling at 100°C for 1 hr. The extract was then filtered through filter paper number 1 (Whatman, Kent, UK) with negative pressure pump (Hicovac, Köln, Germany). The filtrate was subsequently evaporated using lyophilizer (GEA Process Engineering Inc., SC, USA). Lyophilized CGE was stored at 4°C prior to subsequent experiments. The CGE was standardized by determination of total phenolic content using Folin-Ciocalteu reagent similar to that recently described in Rattanapot et al. [20].

The chemical constituents of CGE were further quantitated by high-performance liquid chromatography (HPLC) with diode array detection and mass spectrometry (HPLC-DAD/MSD) method on Agilent Technologies 1100 series (Waldbronn, Germany) equipped with a Zorbax SB C18 column (4.6 mm × 150 mm × 5 *μ*m) and diode array detector recording at 270, 330, 350, and 370 nm. The flow rate was 1 mL/min. The mobile phase was a binary solvent system consisting of acetonitrile (solvent A) and 10 mM ammonium formate (pH 4 with formic acid) (solvent B), with a gradient starting at 0 : 100 (A : B) for 5 min and 20 : 80 for 15 min, and the composition was changed to 40 : 60 over 40 min. Nitrogen was used as nebulizing gas with a flow rate of 13 L/min at 320°C. The capillary voltages were set at 4 kV (positive) and 3 kV (negative). The scan time of 0.2 sec with the range of 100–700* m/z* was used. The chromatographic and spectral features of the CGE were quantitatively analyzed by retention time and peak area under the curve relative to standard of each identified compound.

### 2.3. Oral Glucose Tolerance Test

This experiment was to first address the effective dose of CGE on blood glucose levels with an oral glucose challenge. Normal Wistar rats were fasted overnight and randomly divided into 5 groups. Blood samples of each group were initially collected via tail vein and designated as control base line of blood glucose. The animals were then pretreated with either vehicle (sterile water), CGE dissolved in sterile water at the dose of 0.25, 0.5, and 1.0 g/kg BW, or metformin dissolved in sterile water (20 mg/kg BW) for 30 min. Subsequently, 2.0 g/kg BW of glucose was given in each animal and blood samples were collected every 30 min for 120 min. Blood glucose levels were determined using commercial enzymatic colorimetric assay (Erba Diagnostics Mannheim GmbH, Mannheim, Germany).

### 2.4. Animals and Induction of Experimental Diabetic Rats

Male Wistar rats each weighing 120–150 g were obtained from the National Laboratory Animal Center, Mahidol University, Salaya, Thailand. The animal facilities and protocols were approved by the Laboratory Animal Care and Use Committee at Faculty of Medicine, Chiang Mai University, Chiang Mai, Thailand. All experimental rats were housed in a room maintained at 25 ± 1°C on a 12 : 12 h dark–light cycle. The animals were randomized and divided into 5 groups: normal control (ND), normal supplemented with CGE at the dose of 1.0 g/kg BW (ND + CGE), T2DM (DM), T2DM supplemented with CGE at the dose of 1.0 g/kg BW (DM + CGE), and T2DM treated with antihyperglycemic drug, metformin, at the dose of 30 mg/kg BW (DM + metformin) modified from previous study [21]. For induction condition, the animals were fed with either commercially normal (20% calories as fat) or high fat (58% calories as fat) diets for 2 weeks. Thereafter, rats belonging to DM group were intraperitoneally injected by low-single dose of STZ (40 mg/kg BW) dissolved in 0.1 M citrate buffer while rats in ND group were given citrate buffer as a vehicle as previously described [22]. Ten days after the injection, the overnight fasting blood glucose of all animals was measured from whole blood collected from tail vein by a portable glucometer (Roche Diagnostics Limited, IN, USA) and rats in ND group with fasting blood glucose levels less than 150 mg/dL were designated as ND. Rats belonging to DM group with fasting blood glucose levels exceeding 250 mg/dL were designated as T2DM, while those with the glucose levels below 250 mg/dL were excluded from the study. Subsequently, normal and T2DM rats supplemented with CGE dissolved in sterile water (50 mg/mL) were orally gavage fed 2–4 times daily (at 9 a.m., 12 p.m., 3 p.m., and 6 p.m.) with a maximum volume of 3 mL each time for the subsequent 12 weeks. To prevent any stress to rat caused by gavage feeding, each manipulation was conducted gently, and the feed volume was carefully controlled. Depending on the body weight of the rat, nonetheless, our experiment had only allowed for a maximum total volume of 11 mL for 4 times a day, as the optimal range of a single gavage feeding should not exceed 10 mL/kg as suggested by Brown et al. [23]. In addition, rats in ND, DM, and DM + metformin (30 mg/kg BW) groups which had the same body weight as CGE supplemented rats were additionally dosed with sterile water daily at an equivalent volume for comparable gastric distention in all experimental animals. At the end of study, the animals were sacrificed; blood and tissue samples were collected for subsequent experiments.

### 2.5. Determination of Plasma Glucose, Triglyceride, and Insulin Levels

To determine hyperglycaemia, hyperlipidemia, and insulin concentration, the quantitative total plasma glucose and triglyceride (Biotech Reagent, Bangkok, Thailand) were determined by commercial enzymatic colorimetric assays. The plasma insulin concentration assay was obtained using a Sandwich ELISA assay kit from LINCO Research (Millipore, MA, USA). Insulin resistance index was estimated using the homeostasis assessment of insulin resistance (HOMA index) that was calculated by the following formula: fasting plasma insulin (*μ*U/mL) × fasting plasma glucose (mmol/L)/(22.5).

### 2.6. Histological Examination

To assess renal morphology, the kidneys were cut and one-half of the kidney was fixed in 10% neutral formalin buffer for 12–24 h and then embedded in paraffin. Each slide was cut into 5–7 *μ*m thick sections and stained by Hematoxylin and Eosin (H&E) and the lesions were confirmed by Periodic Acid-Schiff (PAS) base, which are appropriate standard methods for renal biopsy as previously described [24]. Bright-field microscopic evaluation was used to determine tissue morphological changes. The semiquantitative determination of glomerular size, mesangial matrix, and tubular lesions was assessed by a modification method from previous studies [24, 25]. The severity was graded to mild, moderate, and severe for focal changes with less than 25%, 25–50%, and greater than 50% of lesion, respectively.

### 2.7. Renal Slice Preparation and Transport Study

To determine renal secretory process, the PAH uptake mediated rOat1 and 3 in renal cortical slices were examined as previously described [18]. Rat kidneys were removed and placed in oxygenated saline buffer, and renal cortical slices (≤0.5 mm; 5–15 mg, wet weight) were cut with a Stadie-Riggs microtome and maintained in ice-cold oxygenated modified Cross and Taggart buffer containing (mM) 95 NaCl, 80 mannitol, 5 KCl, 0.74 CaCl_2_, and 9.5 Na_2_HPO_4_ (pH 7.4). The renal slices were incubated in 0.25 mL of modified Cross and Taggart buffer containing 5 *μ*M [^3^H]-PAH for 30 min at room temperature. For upregulation of rOat1 and 3 function by insulin stimulation, the slices were preincubated in 0.5 mL of buffer in the absence or presence of 30 *μ*g/mL insulin or other tested compounds (see figure legends in detail) for 30 min and then incubated in 0.25 mL of buffer containing 5 *μ*M [^3^H]-PAH for 30 min at RT. The uptake was stopped by the addition of 1 mL of ice-cold buffer. Slices were then washed, blotted, weighed, dissolved in 0.5 mL of 1 N NaOH, and neutralized with 0.5 mL of 1 N HCl. Scintillation fluid of 9 mL was added and the radioactivity was measured using a Liquid Scintillation Analyzer (PerkinElmer Life Sciences, MA, USA). The uptake of [^3^H]-PAH was calculated as tissue to medium (T/M) ratio, that is, (DPM/g tissue)/(DPM mL medium).

### 2.8. Quantitative Real-Time PCR Analysis

Total RNA was purified from freshly isolated rat renal cortical tissues using total RNA extraction kit (Amresco, OH, USA), according to the manufacturer's instruction. The first strand cDNA was obtained using iScript cDNA synthesis kit (Bio-Rad, CA, USA) and qPCR was performed using SYBR real-time PCR master mix (Toyobo, Osaka, Japan) on ABI 7500 (Life Technologies, NY, USA). Primers were designed according to published sequences (Table 1) and were purchased from Biobasic Inc. (ON, Canada). Gene expressions were normalized to actin mRNA levels and reported as relative fold changes (RFC). QPCR amplification was performed in duplicate for each cDNA.

### 2.9. Subcellular Fractions and Western Blot Analysis

To determine target protein expressions in each cellular compartment, subcellular fractions were extracted from renal cortical tissues using differential centrifugation as previously described [16]. In brief, renal cortical tissues were cut and suspended in CelLytic MT mammalian tissue lysis/extraction reagent (Sigma Aldrich, MO, USA) containing 1% complete protease inhibitor cocktail (Roche Applied Science, IN, USA). The samples were subsequently centrifuged at 5,000 g for 10 min at 4°C. The supernatant was specified as* whole cell lysate* and the pellet was re-suspended in the same buffer and centrifuged at 10,000 g for 10 min at 4°C. The supernatant from this step was specified as* nuclei-rich fraction*. Whole cell lysate fraction was subsequently centrifuged at 100,000 g for 2 h at 4°C and supernatant from the spin was specified as the* cytosolic fraction*. The crude membrane pellets were resuspended in the same solution and specified as* membrane-rich fraction*. The total protein concentration of each sample was measured using commercial Bradford's assay (Bio-Rad, CA, USA) and stored at –80°C prior to use in subsequent experiments.

For western blotting, the protein samples (50 *μ*g/lane for membrane and cytosolic samples and 100 *μ*g/lane for whole cell lysate and nuclei samples) were resolved in 2X Laemmli solution and separated on 10% sodium dodecyl sulfate polyacrylamide gel. The proteins were subsequently transferred onto polyvinylidene difluoride membrane (GE Healthcare, WI, USA) using Bio-Rad system (CA, USA). Nonspecific bindings on the membrane were then eliminated by blocking with 5% nonfat dry milk in 0.05% Tween 20 in Tris-buffered saline (TBS-T) for 1 h at 4°C and subsequently incubated overnight with desired specific primary antibody against PKC*α*, p-PKC*α*, PKC*ζ*, and p-PKC*ζ* (see figure captions). To confirm the enrichment of the fraction, anti-Na^+^-K^+^-ATPase and anti-lamin B1 antibodies were also applied for presenting membrane and nuclei fractions, respectively. Anti-*β* actin antibody was also used as loading control for all samples. The membranes were washed with TBS-T and incubated with goat-anti mouse or rabbit IgG horseradish peroxidase-conjugated secondary antibody (Santa Cruz, CA, USA) for 1 h at 4°C. The target proteins were then detected by enhanced chemiluminescent kit (GE Healthcare, Buckinghamshire, UK) and quantitatively analyzed by Image J program from Research Services Branch (RSB) of the National Institute of Mental Health (NIMH, MD, USA).

### 2.10. Statistical Analysis

Data were expressed as mean ± SD. Statistical differences were assessed using one-way analysis of variance followed by Tukey-Kramer test. Insulin stimulation experiment was analyzed using unpaired, two-tailed Student's *t*-test. Statistical analyses were conducted using Statistical Package for the Social Sciences (SPSS) version 11.5 (SPSS Inc., IL, USA). Differences were considered to be significant when *P* < 0.05.

## 3. Results

### 3.1. Effect of* Cladophora glomerata* Extract on Oral Glucose Tolerance Test in Normal Rats

**Figure 1:**
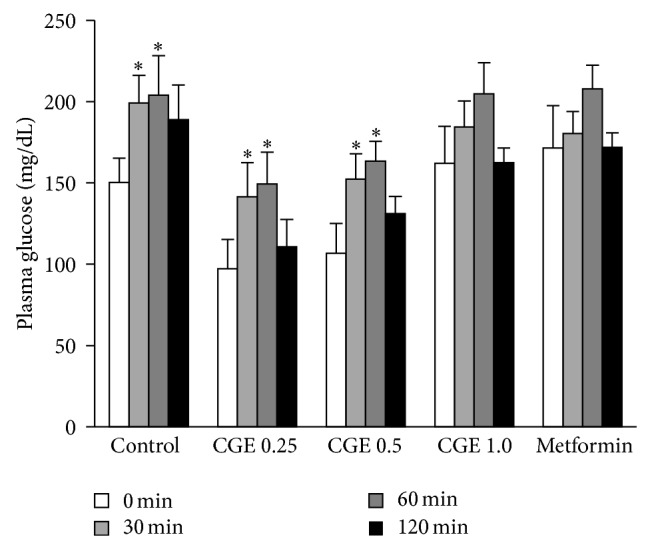
Acute antihyperglycemic effect of* Cladophora glomerata* in normal rats. Oral administration of 0.25, 0.5, and 1 g/kg BW of CGE and 20 mg/kg BW of metformin was given to normal rats for 30 min. Subsequently, 50% glucose solution at a concentration of 2 g/kg BW was administered into each rat. Blood samples were individually collected and blood glucose levels were then determined (*n* = 6–8). ^∗^ *P* < 0.05 indicates significant differences from blood glucose level before treatment.

To determine whether CGE has a potential antidiabetic effect, oral glucose tolerance test was addressed in normal rats. As shown in Figure 1, blood glucose level in control group was significantly increased at 30 and 60 min and tended to return to normal level at 120 min after 2 g/kg BW of glucose loading similarly to that of the animals from pretreatment with CGE at the doses of 0.25 and 0.5 g/kg BW. However, blood glucose levels of the rats pretreated with CGE at a dose of 1.0 g/kg BW or 20 mg/kg BW of metformin did not change at any time after glucose loading, indicating that CGE at 1.0 g/kg BW was able to reduce blood glucose level similarly to that of metformin, an antihyperglycemic drug. Therefore, CGE at the highest dose (1.0 g/kg BW) was applied to the subsequent experiments.

### 3.2. Effects of* Cladophora glomerata *on Type 2 Diabetes Characteristics

The general characteristics of T2DM rats and the effect of CGE on T2DM characteristics were determined. As shown in Table 2, the body weight (BW) and kidney weight per body weight ratio (KW/BW) were not different among experimental groups. The levels of fasting plasma glucose and triglyceride had significantly increased in T2DM rats compared to that of control. In contrast, these plasma parameters were markedly reduced in DM + CGE and DM + metformin compared to that of T2DM rats. Although there was no significant difference in plasma insulin among experimental groups, the HOMA index that indicates insulin resistance had significantly increased in T2DM rats compared to that of control. These HOMA indices were significantly decreased by CGE or metformin treatment relative to that of T2DM. This result suggested that CGE was able to reduce blood glucose and triglycerides and restore insulin resistance after diabetic condition. Moreover, plasma parameters were not altered in ND + CGE compared to that of control, indicating that CGE at the dose of 1.0 g/kg BW/day did not alter plasma glucose and lipid profiles in normal rats.

### 3.3. Effects of* Cladophora glomerata* Extract on Kidney Morphology in T2DM Rats

**Figure 2:**
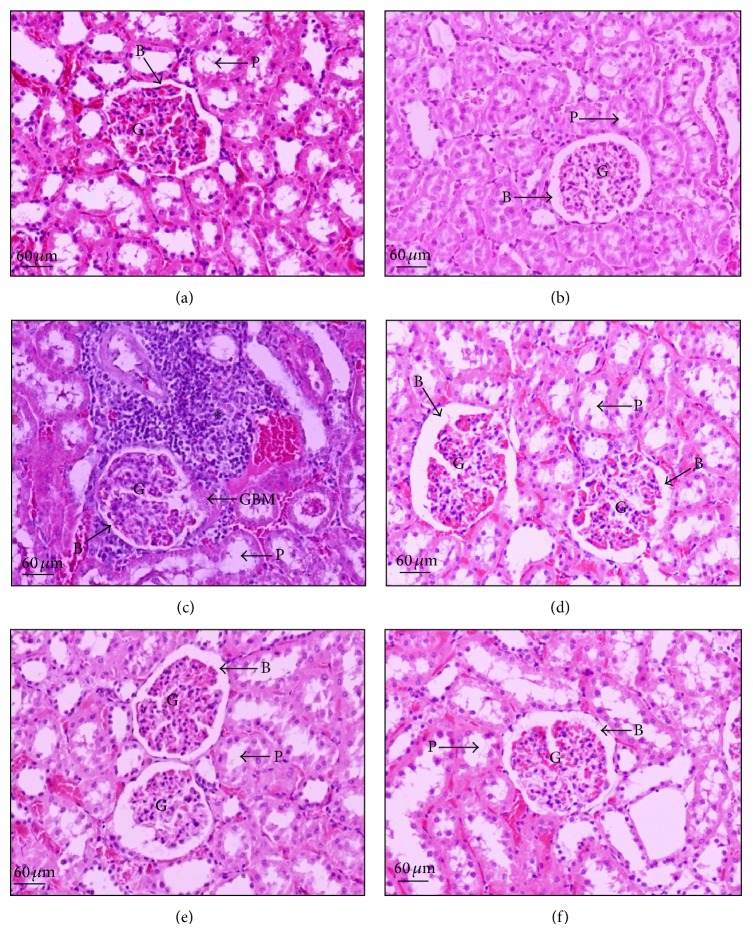
Micrographs of conventional Hematoxylin and Eosin (H&E) staining of rat kidneys. A sagittal half of kidney from each experimental group was removed, fixed, embedded, cut, and stained by H&E (original magnification 200x for all panels). The data were repeated at least 3 times from separate sets of animals. The results were analyzed using bright-field microscopy. (a) Normal (ND), (b) normal diet supplemented with CGE (ND + CGE), (c) T2DM (DM), (d) T2DM treated with metformin (DM + metformin), and ((e) and (f)) T2DM supplemented with CGE (DM + CGE). Arrow “B” in all panels indicates Bowman's capsule space. Arrow labeled “P” indicates the proximal tubular epithelial cells and the tubular lumen space. Asterisk (∗) indicates infiltration of blood cells and lymphocytes. Glomerulus (G), proximal tubular epithelial cells and the tubular lumen space (P), and glomerular basement membrane (GBM).

**Figure 3:**
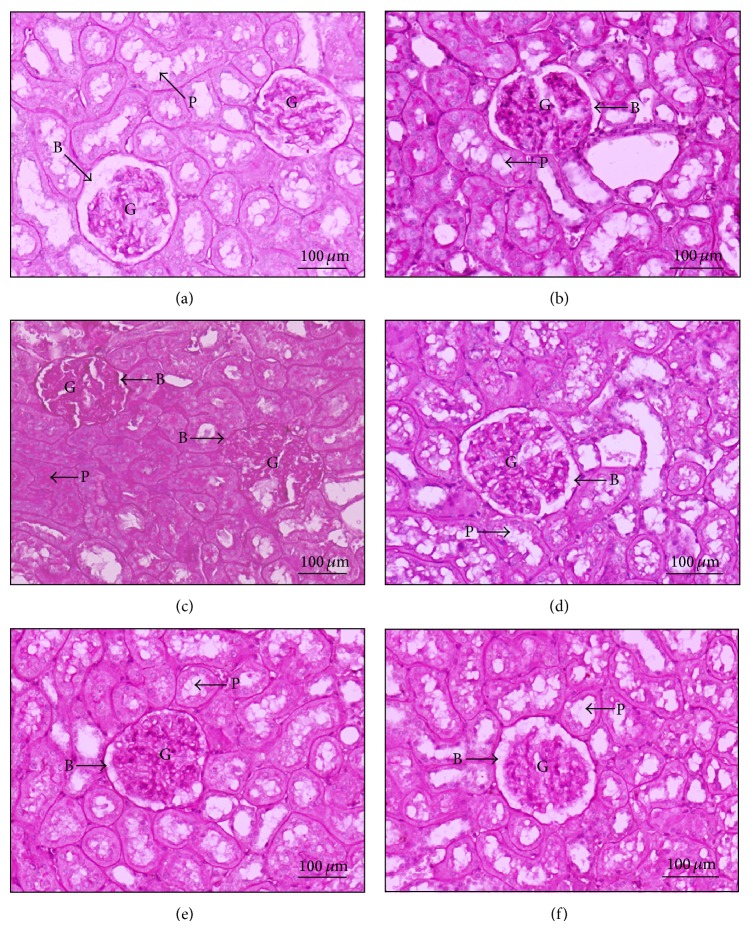
Micrographs of Periodic Acid-Schiff (PAS) staining of rat kidneys. Light microscopies of sagittal half of kidney sections stained with PAS and counterstained with hematoxylin are shown (original magnification 200x for all panels). (a) Normal (ND), (b) normal diet supplemented with CGE (ND + CGE), (c) type 2 diabetes mellitus (DM), and (d) T2DM supplemented with CGE (DM + CGE). Bowman's capsular spaces are indicated by arrow “B”; the proximal tubular epithelium and the lumen are indicated by arrow “P.” Glomerulus (G).

To evaluate the effect of CGE on renal structural changes in T2DM rat model, renal morphological analysis was semiquantitatively assessed using standard methods for renal biopsy, Hematoxylin and Eosin (H&E; Figure 2), and Periodic Acid-Schiff (PAS; Figure 3) base stains. As shown in Figure 2(a), normal diet fed rats (ND) had normal renal structures including glomerulus, Bowman's capsule space, and proximal convoluted tubules similar to that of ND + CGE rat kidneys (Figure 2(b)). On the other hand, T2DM rat kidney markedly exhibited the diffused white blood cell infiltration in the interstitial space and a glomerular hypertrophic formation with a narrowing of Bowman's capsule space (Figure 2(c), black arrow). In addition, the interstitial space was densely infiltrated with lymphocytes and blood cells, and mild arteriolar hypertrophy was observed. The similar pattern of infiltration was also seen in the tubulointerstital area (Figure 2(c), asterisk). The histological changes of DM + metformin appeared to have recovered since they closely matched normal control, as seen by a decrease of glomerular capillaries and tubular hypertrophy. Nevertheless, there was a mild infiltration of red blood cells and lymphocytes in the tubulointerstitium. In addition, Bowman's capsule space and tubular lumen of DM + metformin were wider than T2DM alone (Figure 2(d) versus Figure 2(c)). In contrast, the glomeruli and tubular cells of DM + CGE showed progressively reduced infiltration and mild arteriolar changes (Figures 2(e) and 2(f)). Compared to T2DM, the glomeruli structures in DM + CGE were recovered, as evidenced by the presence of Bowman's capsule and tubular luminal spaces similarly to that of DM + metformin. Therefore, this result indicated that CGE improved in both glomerular infiltration and tubular hypertrophy in renal tissues similarly to that of metformin treatment.

In the PAS staining, T2DM rat kidneys demonstrated the moderate lesion of mesangium, glomerular basement membrane thickening, and a narrow renal tubular lumen (Figure 3(c)) compared to that of ND and ND + CGE (Figures 3(a) and 3(b)). Moreover, Bowman's capsule of T2DM rat kidneys was intensively stained, implying that basement membranes were thickening and the production of extracellular matrix was increased in T2DM. In contrast, the kidney morphology of DM + CGE was very similarly to that of control (ND; Figure 3(a)), including the reduction in the extent of the glomerular hypertrophy and mesangial expansion (Figure 3(d)). As a result, the tubular lumen and Bowman's capsule spaces were seen (Figure 3(d), arrow P and B). Similar to DM + CGE, renal tissues of DM + metformin displayed the improvement of mesangial and tubular hypertrophy. Hence, this study clearly demonstrated that T2DM rats supplemented with CGE at the dose of 1.0 g/kg BW manifested reduced adverse morphological changes, and there was no deleterious effect in kidneys in normal animals dosed with CGE (ND + CGE).

### 3.4. *Cladophora glomerata *Did Not Alter rOat1 and rOat3 Functions but Rather Improved Insulin-Stimulated PAH Transport in T2DM Rats

**Figure 4:**
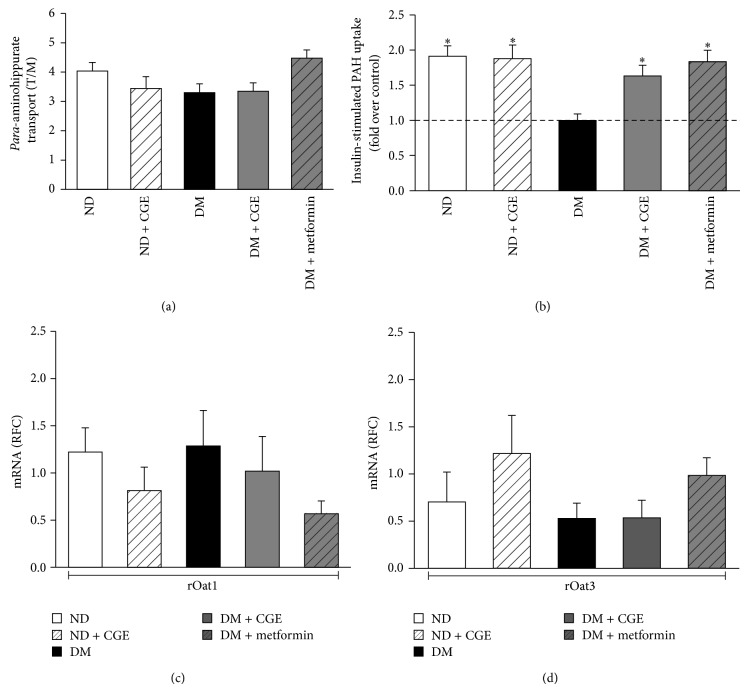
Effects of* Cladophora glomerata *on PAH transport by rOat1 and rOat3 and their mRNA expressions. (a) Rat renal cortical slices were incubated for 30 min in the buffer containing 5 *μ*M of [^3^H]-PAH at room temperature. Data are expressed as tissue to medium ratios (T/M), that is, tissue content (DPM/mg) ÷ medium (DPM/*μ*L), and represented as mean ± SD. Each experiment was performed from separate animals and at least 5 renal slices were used in each condition (*n* = 6–8). (b) Rat renal cortical slices were preincubated for 30 min in the presence or absence of 30 *μ*g/mL of insulin and followed by incubation with classical substrate of organic anion transporters, 5 *μ*M of [^3^H]-PAH for 30 min. Data are expressed as fold over control (without insulin). Each experiment was performed from separate sets of animals and 5 renal slices were used in each condition (*n* = 6). ^∗^ *P* < 0.05 indicates significant differences from the slices incubated with buffer alone. (c) Rat Oat1 and (d) Rat Oat3 mRNA expressions in renal cortical tissues. Total RNAs were extracted from rat cortical tissues from each animal and rOat1 and 3 mRNA levels were measured using quantitative real-time PCR (qPCR). The data are expressed as mean ± SD and repeated from separate animals (*n* = 5–7).

As mentioned earlier, renal Oat1 and 3 were influenced by certain pathological status and strongly correlated with the progression of diabetic kidney diseases [16] and* para*-aminohippurate (PAH) is a prototypical substrate for rOat1 and 3 transporters that are mainly localized on the basolateral membrane of renal proximal tubules [8, 9, 11]. We subsequently investigate the impact of T2DM on renal organic anion transport function using renal cortical slices. Thus, we conducted functional transport assay using [^3^H]-PAH uptake into renal cortical slices. As shown in Figure 4(a), PAH uptake in renal cortical slices was not different among experimental groups. This result indicated that T2DM status and T2DM supplemented with CGE in rats had no effect on basal renal organic anion transport function mediated by rOat1 and 3.

Since the basal physiological rOat1 and 3 functions did not change by T2DM status and CGE supplementation, we further addressed whether CGE had any effects on the regulatory proteins for rOat1 and 3 functions. A study by Barros et al. [18] had shown that rOat1 and 3 functions were upregulated after insulin preincubation. We, therefore, determined the regulatory function of rOat1 and 3 using [^3^H]-PAH uptake in rat renal cortical slices in the presence or absence of 30 *μ*g/mL of insulin preincubation. As shown in Figure 4(b), the slices from ND rats that were preincubated with the insulin had almost two-fold significant increase in [^3^H]-PAH transport compared to that of slices without insulin, indicating that upregulated rOat1 and 3 functions by insulin were reproducible similar to that of the previous study [18]. On the other hand, the effect of insulin on [^3^H]-PAH uptake was blunted in renal slices of T2DM rats, suggesting that T2DM rat kidneys had significantly impaired insulin-stimulated rOat1 and 3 functions. Interestingly, the effect of insulin stimulation on the increase of PAH transport remained present in the renal slices from ND + CGE, DM + CGE and DM + metformin similarly to the slices from control, implying that 1 g/kg BW of CGE was able to improve regulatory function of rOat1 and 3 similarly to that of metformin treatment in T2DM rats.

### 3.5. *Cladophora glomerata* Did Not Alter rOat1 and rOat3 Gene Expressions in Renal Cortical Tissues

To further clarify the mechanism by which CGE improved upregulation of rOat1 and 3 functions, we investigated whether gene transcripts for these transporters were modulated by CGE under diabetic condition, leading to a restoration of rOat1 and 3 functions. Thus, quantitative real-time PCR was performed using rat renal cortical tissues. Genes encoding for rOat1 and 3 mRNA were subsequently determined. As shown in Figure 4(c), rOat1 and 3 mRNA expressions were not different among experimental groups. Therefore, this result indicated that the restoration of regulatory mechanism of rOat1 and 3 after CGE supplementation in T2DM rats was not the result of changes of rOat1 and 3 gene expressions but involves other mechanisms.

### 3.6. Effect of* Cladophora glomerata* on Classical PKC Expression and Activation in Renal Cortical Tissues

**Figure 5:**
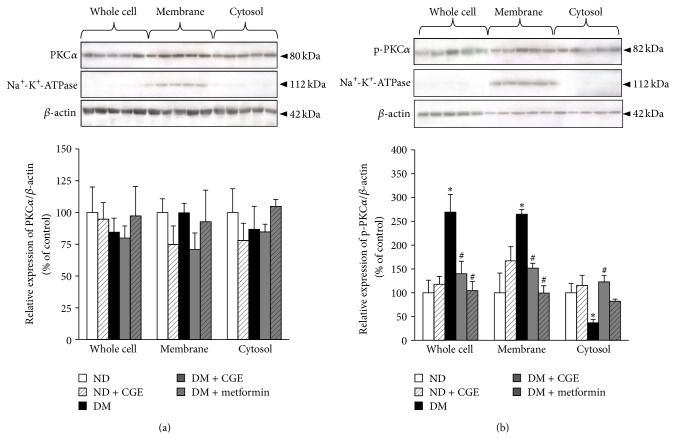
Effects of* Cladophora glomerata* extract on PKC*α* expression, activation, and translocation in renal cortical tissues. Whole cell lysate, cytosolic, and nuclei fractions were extracted from rat renal cortical tissues. The samples were then separated using electrophoresis and western blotting. (a) Anti-PKC*α* and (b) p-PKC*α* antibodies were subsequently detected while anti-Na^+^-K^+^-ATPase and anti-*β*-actin antibodies were used as a membrane marker and loading control, respectively. The data are expressed as mean ± SD and repeated from separate sets of animals (*n* = 5). A representative blot of PKC*α* and p-PKC*α* protein expressions is shown in* top panel *((a) and (b)) and quantification of relative protein expression in each fraction is presented in* bottom panel *((a) and (b)).

Activation of classical PKCs isoforms has previously shown to be a predominant signaling mediator in progressive development of diabetic nephropathy [4, 5]. In addition, rOat3 function was downregulated by PKC*α* activation, and its function was reversed by GÖ6976, a specific PKC*α* inhibitor [17]. Thus, we then identified whether CGE improved rOat1 and 3 functions through specific PKC*α* expression and function. Subcellular fractions extracted from renal cortical tissues and western blotting analysis were subsequently carried out. The detection of PKC*α* and p-PKC*α* proteins was analyzed by densitometry and the amount of each target protein in each sample was normalized by the amount of *β*-actin present in each respective fraction. As shown in Figures 5(a) and 5(b), Na^+^K^+^-ATPase, a basolateral membrane marker, was detected only in membrane fraction of each experimental group. Despite that, total PKC*α* expressions were not significantly different in whole cell lysate, membrane, and cytosolic fractions among experimental groups (Figure 5(a)). In contrast to total PKC*α*, activated PKC*α* (p-PKC*α*) were markedly increased in both whole cell and membrane fractions in T2DM rat kidneys while it was significantly reduced in cytosolic fraction, indicating that T2DM status resulted in the activation and translocation of PKC*α* to the plasma membrane (Figure 5(b)). Interestingly, p-PKC*α* were significantly reduced in both whole cell and membrane fractions in DM + CGE and DM + metformin rat kidneys relative to that of T2DM, suggesting that CGE and metformin partially prevented PKC*α* activation and translocation to the membrane under T2DM condition (Figure 5(b)). Moreover, there was no significant difference of p-PKC*α* in any fraction of ND + CGE rat kidneys compared to that of ND, indicating that CGE did not alter intracellular PKC*α* protein expression and activation under normal condition.

### 3.7. *Cladophora glomerata* Modulated Protein Kinase C*ζ* Activity and Translocation in Renal Cortical Tissues

**Figure 6:**
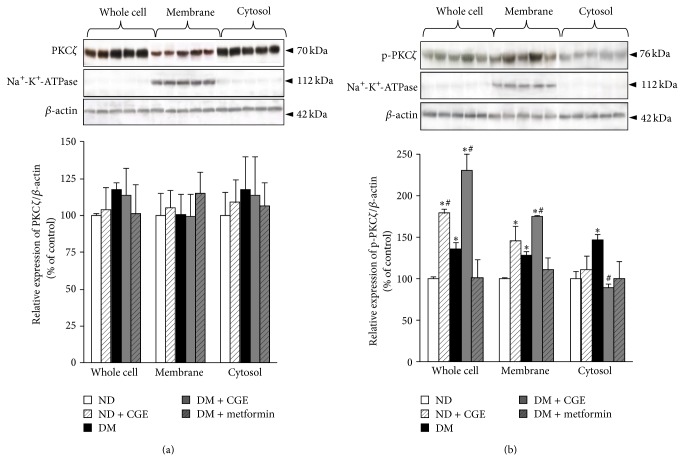
Effects of* Cladophora glomerata* extract on PKC*ζ* expression, activation, and translocation in renal cortical tissues. Whole cell lysate, cytosolic, and nuclei fractions were extracted from rat renal cortical tissues. The samples were then separated using electrophoresis and western blotting. (a) Anti-PKC*ζ* and (b) p-PKC*ζ* antibodies were subsequently detected while anti-Na^+^-K^+^-ATPase and anti-*β*-actin antibodies were used as a membrane marker and loading control, respectively. The data are expressed as mean ± SD and repeated from separate sets of animals (*n* = 5). A representative blot of PKC*ζ* and p-PKC*ζ* protein expressions is shown in* top panel *((a) and (b)) and quantification of relative protein expression in each fraction is presented in* bottom panel *((a) and (b)).

Our previous data demonstrated that CGE prevented PKC*α* activation and membrane translocation under T2DM status, which resulted in partially restored insulin-stimulated PAH transport mediated by rOat1 and 3 (Figure 5). We also addressed whether CGE improved rOat1 and 3 regulatory functions by directly modulating their stimulatory proteins, PKC*ζ*, using western blotting analysis. The detection of PKC*ζ* and p-PKC*ζ* proteins was analyzed by densitometry and the amount of each target protein in each sample was normalized by the amount of *β*-actin present in each respective fraction as shown in Figure 6. Consistently, Na^+^K^+^-ATPase was detected only in membrane samples of each experimental group, and total PKC*ζ* was not significantly different in any fraction of experimental groups (Figure 6(a)). However, activated PKC*ζ* (p-PKC*ζ*) in whole cell lysate, membrane, and cytosolic fractions were significantly increased in T2DM relative to control, suggesting that diabetic condition partially influenced PKC*ζ* activity (Figure 6(b)). In addition, p-PKC*ζ* also significantly increased in whole cells and membrane fractions in both ND + CGE and DM + CGE rat kidneys compared to that of ND, which strongly indicated that, besides diabetic status, CGE supplementation also induced PKC*ζ* activation and its membrane translocation while the direct effect of metformin on p-PKC*ζ* expression was not observed. Interestingly, the significant increase of p-PKC*ζ* in membrane fraction of DM + CGE was higher than T2DM, suggesting that not only was there diabetic status, but CGE also exerted PKC*ζ* activity, too. Nonetheless, a decrease of p-PKC*ζ* in cytosolic fraction of DM + CGE was only seen relative to T2DM. This data suggested that CGE stimulated PKC*ζ* and subsequently induced membrane translocation of p-PKC*ζ*, resulting in restoration of rOa1 and 3 regulatory functions under the T2DM status (Figure 6(b)).

### 3.8. *Cladophora glomerata* Extract Directly Modulated Protein Kinases Activities in Renal Cortical Tissues

**Figure 7:**
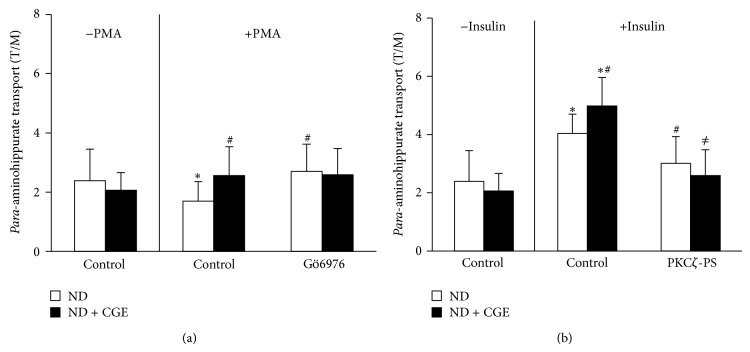
Effects of* Cladophora glomerata* extract on regulatory function of rOat1 and rOat3 functions. (a) Downregulation of rOat1 and 3 by PMA-inhibited PKC*α* activity. Rat renal cortical slices were preincubated for 30 min in the presence or absence of 100 nM of PMA or PMA with GÖ6976 and followed by incubation with 5 *μ*M of [^3^H]-PAH for 30 min. Data are expressed as tissue to medium ratios (T/M) (*n* = 5). ^∗^ *P* < 0.05 indicates significant differences from control. ^#^ *P* < 0.05 indicates significant differences from PMA preincubation in renal slices from ND. (b) Upregulation of rOat1 and 3 by insulin-stimulated PKC*ζ* activity. Rat renal cortical slices were preincubated for 30 min in the presence or absence of 30 *μ*g/mL of insulin or insulin with PKC*ζ*-PS and followed by incubation with 5 *μ*M of [^3^H]-PAH for 30 min. Data are expressed as tissue to medium ratios (T/M) (*n* = 5–7). ^∗^ *P* < 0.05 indicates significant differences from respective control. ^#^ *P* < 0.05 indicates significant differences from insulin preincubation in renal slices from ND. ^≠^ *P* < 0.05 indicates significant differences from insulin preincubation in renal slices from ND + CGE.

According to the above findings, previous study indicated that angiotensin II activated specific PKC*α*, leading to downregulation of Oat3 function, but GÖ6976, the PKC*α* inhibitor, specifically reversed its effect [17]. In addition, insulin stimulated PKC*ζ*, resulting in upregulated Oat1 and 3 functions, and a PKC*ζ* inhibitor, PKC*ζ*-PS, significantly reverted insulin's effect [18]. Therefore, we further clarified if CGE could act directly through both PKC*α* and PKC*ζ* activities mediated rOat1 and 3 signaling pathway using renal slices from ND and ND + CGE rats. Preincubation of PMA, a classical PKC activator, inhibited PAH transport mediated by rOat1 and 3 in the slices from ND, whereas GÖ6976 restored this inhibitory effect (Figure 7(a)). Interestingly, the slices from ND + CGE displayed no difference in PAH uptake after PMA preincubation in any condition, suggesting that CGE directly prevented the activation of PKC*α* activity, resulting in maintaining the functional transport of PAH mediated by rOat1 and 3. Unlike PKC*α* activity, preincubation of insulin significantly stimulated PAH uptake in ND rat renal slices while PKC*ζ*-PS inhibited the insulin effect (Figure 7(b)). Again, the slices from ND + CGE showed an insulin-activated PKC*ζ* activity and an increase in PAH uptake. PKC*ζ*-PS was also able to inhibit PAH uptake after insulin stimulation similarly to that of the slices from ND (Figure 7(b)). Hence, this result strongly indicated that CGE directly enhanced PKC*ζ* activity, leading to continuation of the insulin-stimulated rOat1 and 3 functions.

### 3.9. Bioactive Compounds in* Cladophora glomerata* Extract

**Figure 8:**
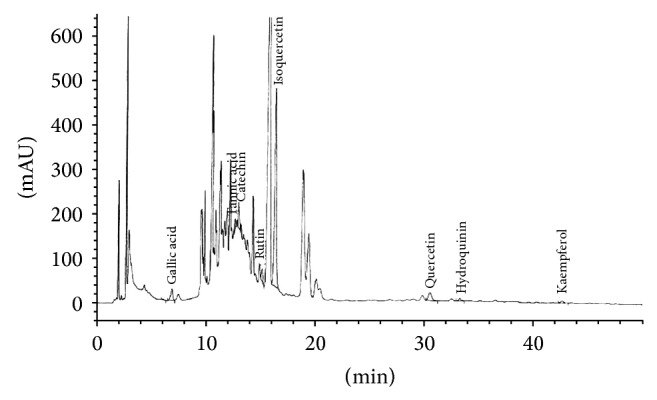
*Cladophora glomerata* extract qualification using HPLC-DAD/MSD analysis. CGE was analyzed against their phenolic standards. Semiquantitative data was analyzed by peak area under the curve relative to the content of each component in the extract.

After* Cladophora glomerata* was extracted, the total phenolic content was subsequently determined. The data showed that the total phenolic content was 8.36 ± 0.13 mg gallic acid equivalent/g of extract. In addition, the major chemical constituents of CGE were also quantitated by HPLC-DAD/MSD (Figure 8). Several phenolic contents including catechin, tannic acid, hydroquinin, quercetin, rutin, gallic acid, and kaempferol were found at the amount of 2.2, 1.3, 1.1, 1.0, 0.63, 0.58, and 0.14 g/kg of extract compared to that of each respective standard. Among these, isoquercetin (7.9 g/kg of extract) was shown to be the highest constituent compared to that of respective standards.

## 4. Discussion

*Cladophora glomerata* (CG) Kützing has been widely grown in Nan River, north of Thailand. This alga contains high amount of carbohydrate, fat, proteins, multivitamins, and minerals. A recent study has demonstrated that the aqueous extract of CG (CGE) contains phenolic contents that exhibited antigastric ulcer, analgesic, hypotensive, anti-inflammatory, and antioxidant activities both* in vivo* and* in vitro*, suggesting that CGE has potential to be developed as nutraceutical products [1]. Moreover, neither a high dose (25 g/kg BW) nor subchronic (up to 1 g/kg BW for 60 days) administration of CGE showed toxicity in any organs in rats [26]. Yet, the therapeutic effects of CGE on a particular disease have not been reported previously. To address the potential use of CGE, this study investigated the pharmacological effects of CGE under diabetes status and clearly demonstrated that CGE has antihyperglycaemia, antihypertriglyceridemia, anti-insulin resistance, and renoprotective effects in an experimental model of type 2 diabetes in rats. As summarized in Table 2, the significant increase in the levels of fasting plasma glucose, triglyceride, and HOMA index found in our experimental T2DM rat model was similar to those observed in human. In addition, the crude CGE at the dose of 1 g/kg BW had antidiabetic activities similar to treatment by an antihyperglycemic drug, metformin. Nevertheless, further studies on its purified potential constituents are required for future development of CGE into a pharmaceutical or nutraceutical product for diabetes. Similarly to CGE,* Spirogyra neglecta* Kützing extract, one of the macroalgae, had recently shown antihyperglycaemia, antihyperlipidemia, and improved insulin resistance in T2DM rats [18, 27]. Moreover,* Aloe vera* leaves extract has also demonstrated hypoglycemic effect in both T1DM and T2DM rats [28], and the quercetin-rich onion peel extract had shown to improve hyperglycaemia and insulin sensitivity by upregulation of insulin receptor and glucose transporter 4 mRNA expressions [29]. Hence, the precise mechanism by which CGE ameliorated hyperglycaemia and hypertriglyceridemia and restored the whole body insulin sensitivity in T2DM requires further investigation.

Diabetic nephropathy is a major cause of end-stage renal disease, and its progression cannot always be completely accurately predicted by clinical parameters, especially in early stages [24, 25]. Renal pathological classifications and diagnoses, as performed in this study, allowed us to predict the changes in renal morphology in our T2DM rat model. As shown in PAS staining, the thickening of glomerular and tubular basement membrane of T2DM rat kidneys indicated the impairment in “autoregulation” of glomerular microcirculation that might consequently affect glomerular filtration rate. In addition, the presence of mesangial expansion in T2DM rat kidney might also influence membrane permeability, which then resulted in a migration of white blood cells from capillary endothelium to tubular interstitial as previously shown [24, 25]. Taking these into account, we have shown that CGE was able to completely reverse the progressive loss of glomerular and tubular permeability and functions. Similarly to CGE, traditional Chinese medicine Xiao-Chai-Hu-Tang was found to restore mesangial expansion, basement membrane thickening, glomerular hypertrophy, and tubular damage in T1DM rats [30] while quinapril, the angiotensin-converting enzyme inhibitor, was also able to restore defective glomerular albumin permeability in experimental diabetic nephropathy rats [31].

The modulations of membrane protein expressions and activities have been extensively shown in several pathological conditions. For example, decreases of Na^+^K^+^-ATPase and glucose transporter in liver, brain, and heart tissues were observed, whereas the expressions of these two transporters were increased in the kidneys in experimental T1DM rats [32]. Moreover, oxonic and uric acids induced hyperuricemia in rats had shown a decrease in renal basolateral PAH uptake, corresponding to the reduction of rOat1 and 3 mRNA and protein expressions [33]. Furthermore, a recent study showed the reduction of Oat1 expression with a decrease of renal organic anion secretion in CRF in rats which altered renal organic anion drug clearances and might exert the accumulation of uremic toxins as seen in CRF in human [34]. Likewise, our recent study indicated that expression of mOat3, but not mOat1, was decreased in parallel with its function in T1DM models, and insulin treatment was able to improve their functions [14]. In addition, previous study suggested that Oat1 and 3 play important role in renal tubular uptake of the retention of waste products, the uremic toxins, for example, indoxyl sulfate, hippurate, indoleacetate, and 3-carboxy-4-methyl-5-propyl-2-furanpropionate, and aggravated tubular damage in renal failure [35]. Hence, the certain levels of Oat1 and 3 expressions and transport activities are required under the progression of renal diseases. More recently, patients with kidney biopsy-proven diabetic nephropathy have demonstrated a 50% downregulation of proximal tubular Oat1 and 3 mRNA expressions. Using metabolomics analysis, this evidence correlated with a decrease in urinary organic anion metabolite makers seen in patients with diabetic kidney disease including homovallinic acid and 4-OH hippurate, supporting that the transport function of Oat1 and 3 was the rate-limiting step and has contributed to the urine metabolomics that could serve as novel biomarkers for diabetes with chronic kidney disease [16]. Although there was no difference in the basal uptake of PAH mediated by rOat1 and 3 in renal cortical slices from T2DM rats in our study, we have shown that insulin-stimulated PAH transport was blunted and might possibly alter other organic anion clearances, and CGE was able to restore insulin effect on PAH transport to levels of normal rat kidneys, implicating that CGE could certainly preserve anionic secretory process in T2DM condition (Figure 4). Indeed, we have primarily shown that neither renal rOat1 nor rOat3 mRNA levels were directly altered by either T2DM or CGE. Hence, modulation of rOat1 and 3 activities through intracellular signaling proteins could be the potential mechanism for CGE-improved organic anion transport in T2DM condition.

Several molecular mechanisms involving hyperglycaemia-induced diabetic nephropathy have been proposed [5, 36]. For instance, hyperglycaemia-induced reactive oxygen species production resulted in activation of PKC and NF*κ*B which, in turn, induced proinflammatory cytokines, TNF*α* and IL1*β* [5, 36, 37]. In addition, the activation of specific PKC*α*, *ε*, and *β* isoforms contributed in diabetic nephropathy rats [38–40] while a high glucose-stimulated PKC*β* was linked with NF*κ*B activation in rat glomerular mesangial cultured cells [41]. Compared to T2DM, our data demonstrated that CGE prevented PKC*α* and induced PKC*ζ* activations and translocation to the plasma membrane of renal epithelial cells (Figures 5 and 6). Certainly, this data cannot preclude the initial biological effects of CGE, the antihyperglycaemia, antihypertriglyceridemia, and anti-insulin resistance, which exerted the subsequent inhibitory and stimulatory effects on PKCs in T2DM rat kidneys. Since PKC comprises of several isoforms and plays a crucial role in several cellular events, their contribution in organic anion transporter functions has been also specified. A previous study also found that Oat3 was downregulated through the activation of specific PKC*α*, and GÖ6976, a specific PKC*α* inhibitor, reversed this consequence [17]. In addition, recent study indicated that rOat1 and 3 were upregulated by insulin-activated PKC*ζ* isoform, and insulin effect was blunted by PKC*ζ*-PS [18]. We then addressed the possibility of whether the improved regulatory functions of rOat1 and 3 by CGE were directly associated with the activation of either PKC*α*, *ζ*, or both isoforms without influence of T2DM condition. Consistently, the present data strongly demonstrated that CGE directly inhibits PKC*α* and stimulates PKC*ζ*, resulting in restoration of insulin-stimulated rOat1 and 3 functions in renal cortical slices similarly to normal rat kidneys (Figure 7). Thus, CGE might also delay or prevent progressive renal structural and functional changes by PKC activities in T2DM rats. However, a thorough understanding of the molecular consequences of CGE actions on renal organic anion transport function in T2DM should be further investigated. In this study, the major phenolic contents included catechin, tannic acid, hydroquinin, quercetin, rutin, gallic acid, kaempferol, and particularly isoquercetin and were identified in CGE. Thus, these constituents might exert or play role as antidiabetic and renoprotective effects in T2DM rats through regulation of PKCs. Likewise, quercetin-rich onion peel extract exhibited antihyperglycaemia and antihyperlipidemia in T2DM rats [29] while isoquercetin demonstrated antidiabetic effect in noninsulin-dependent diabetic or diabetic KK-A^Y^ mice [42]. Indeed, polyphenol-rich extracts from* Solanum nigrum* inhibited HepG2 cell migration by reducing PKC*α* expression and activation [43], and isoquercetin has shown to inhibit liver cancer cell proliferation via direct downregulation of PKC mRNA and protein expressions [44].

In conclusion, the present study demonstrated that CGE has antidiabetic and renoprotective effects in T2DM by restoration of pathogenic consequences, including antihyperglycaemia, antihypertriglyceridemia, anti-insulin resistance, and restoration of insulin stimulated renal rOat1 and 3 functions. The mechanisms by which CGE-enriched polyphenols improved insulin-stimulated rOat1 and 3 functions were initially associated with antidiabetic effects and directly linked with the inhibition of PKC*α* and stimulation of PKC*ζ*, which are responsible for rOat1 and 3 functions. While promising, further data is needed for development of CGE into either a nutraceutical or pharmaceutical product for the prevention of diabetic nephropathy.

## Figures and Tables

**Table 1:** Primer sequences and expected amplicon sizes for the gene amplification.

cDNA	Genbank Acc. number	Forward primer	Reverse primer	Amplicon size (bp)
rOat1	NM017224	CATTGATGGC TGGGTCTATG	CAAGGTTCCACT CAGTCACG	65
rOat3	NM031332	ATCTCATCAACA TCTATTGGGTACTG	CAGAGAGAGACA GAAGGTCACAC	371
*β*-actin	NM031144	ATGGTGGGTA TGGGTCAGAA	GGGGTGTTGAAG GTCTCAAA	241

rOat1: rat organic anion transporter 1; rOat3: rat organic anion transporter 3.

**Table 2:** General characteristics and plasma parameters of experimental rats at the end of week 12.

	ND	ND + CGE	DM	DM + CGE	DM + metformin
General characteristic					
BW (g)	430.0 ± 26.5	420.5 ± 20.3	456.3 ± 30.9	426.7 ± 30.6	416.7 ± 66.6
KW/BW	5.7 ± 0.8	6.0 ± 0.7	5.6 ± 0.9	6.2 ± 1.0	5.9 ± 1.0
Plasma parameter					
Glucose (mg/dL)	135.1 ± 19.9	128.3 ± 15.6	301.0 ± 20.2^*^	149.2 ± 53.8^#^	164.8 ± 38.0^#^
Triglyceride (mmol/L)	140.4 ± 44.4	121.5 ± 24.7	286.7 ± 49.5^*^	142.6 ± 32.0^#^	156.5 ± 40.9^#^
Insulin (ng/mL)	2.8 ± 1.1	2.3 ± 0.4	1.8 ± 0.9	1.7 ± 0.8	1.1 ± 0.7
HOMA index	16.4 ± 4.1	13.4 ± 4.8	28.4 ± 15.1^*^	12.7 ± 4.6^#^	15.9 ± 2.6^#^

Data are presented as mean ± SD from 6 to 8 animals per group. BW: body weight; KW: kidney weight; HOMA: homeostatic model assessment of insulin resistance; ^*^ *P* < 0.05 indicates the significant differences from normal (ND) and ^#^ *P* < 0.05 indicates the significant differences from T2DM (DM) rats.

## Abbreviations

CGE: * Cladophora glomerata* extract
T2DM: Type 2 diabetes mellitus
Oat1: Organic anion transporter 1
Oat3: Organic anion transporter 3
CRF: Chronic renal failure
BW: Body weight
mRNA: Messenger ribonucleic acid
PKC*α*: Protein kinase C alpha
PKC*ζ*: Protein kinase C zeta
PKC*ζ*-PS: Protein kinase C zeta pseudosubstrate
PAH: * para*-aminohippurate
STZ: Streptozotocin
Oct1: Organic cation transporter 1
Oct2: Organic cation transporter 2
mOat3: Mouse organic anion transporter 3
H&E: Hematoxylin and Eosin
PAS: Periodic Acid-Schiff base
PVDF: Polyvinylidene difluoride membrane
TBS: Tris-buffer saline
ECL: Enhanced chemiluminescence
PCR: Polymerase chain reaction
cDNA: Complementary deoxyribonucleic acid
qPCR: Quantitative polymerase chain reaction.
